# Editorial for the Special Issue “Emergency Medicine and Emergency Room Medical Issues II”

**DOI:** 10.3390/medicina60040530

**Published:** 2024-03-25

**Authors:** Carmine Siniscalchi, Egidio Imbalzano, Pierpaolo Di Micco

**Affiliations:** 1 Internal Medicine Unit, Department of Internal Medicine, University of Parma, 43100 Parma, Italy; siniscalchi.carmine@gmail.com; 2 Internal Medicine Unit, Department of Clinical and Experimental Medicine, University of Messina, 98100 Messina, Italy; egidio.imbalzano@unime.it; 3 AFO Medica, UOC Medicina Interna, P.O. Santa Maria delle Grazie, ASL Napoli 2 Nord, 80076 Pozzuoli, Italy

The variety of clinical issues presented by patients, along with the need for a rapid diagnosis and treatment, represents the main reasons for the risk of burnout among physicians who work in emergency departments. Working as a Medical Doctor (MD) in the emergency room is one of the most complex types of role experienced in the health system. We re-proposed a previous Special Issue of *Medicina*, entitled “Emergency medicine and emergency room medical issues II”, due to the success of the previous edition, which can be attributed to the great interest in this topic. Physicians around the world were invited to contribute papers regarding any type of clinical experience in this field.

This Special Issue was born near the SARS-CoV-2 era, and for these reasons, several articles focus in part on aspects of this disease, but not only on these aspects. The management of emergency rooms during the outbreak changed all around the world, such as the management of acute appendicitis during and before the COVID-19 pandemic [1], rather than the management of pediatric [2] and adult emergencies such as hypoxemic respiratory failure during SARS-CoV-2 infection [3]. Additionally, emergency departments adapted their activities to identify and manage the severe complications of vaccinations against SARS-CoV-2, such as vaccine-induced ph-positive B-Cell acute lymphoblastic leukemia occurring after receipt of the bivalent SARS-CoV-2 mRNA vaccine booster [4].

However, acute illnesses affecting non-COVID-19 areas are more fascinating than the usual conditions, such as disorders related to the management of electrolytes [5], versus the most rare, such as a reported case of bilateral traumatic testicular dislocation [6].

Of course, bleeding treatment strategies also occupy a relevant place in research conducted by scholars around the world. The evaluation of the Hitit Index in differential diagnosis of Crimean–Congo hemorrhagic fever in the emergency department [7], the effectiveness of intranasal analgesia in the emergency room [8], and the use of Aflibercept for gastrointestinal bleeding in Hereditary Hemorrhagic Telangiectasia [9] were described.

Another aspect is that the management of and increase in survival rate are still dependent on the speed of triage systems and number of physicians and nurses available in the specific moment. In this sense, authors suggest the use of motorcycle paramedics for patients after out-of-hospital cardiac arrest [10].

Trauma also represents a significant area of hard work in emergency departments. The usefulness of the diagnostic benefit of computed tomography in pediatric cases with multiple traumas is a challenge, and was presented in one of the accepted articles [11].

Finally, in this Special Issue, authors remarked on a crucial aspect of medicine, namely the management of end-of-life care in the emergency department [12], which remains an ever actual and difficult aspect to manage.
